# OTEH-3. Targeted Gene-Expression analysis during malignant transformation in primary and secondary malignant meningioma

**DOI:** 10.1093/noajnl/vdab070.042

**Published:** 2021-07-05

**Authors:** Andrea Daniela Maier, Alessandra Meddis, Jeppe Haslund-Vinding, Christian Mirian, Ausrine Areskeviciute, Phuong Nguyen, Casper Westergaard, Linea Cecilia Melchior, Tina Nørgaard Munch, Jane Skjøth-Rasmussen, Lars Poulsgaard, Morten Ziebell, Jiri Bartek, Helle Broholm, Frantz Rom Poulsen, Thomas Alexander Gerds, David Scheie, Tiit Illimar Mathiesen

**Affiliations:** 1 Rigshospitalet, Department of Neurosurgery, Copenhagen, Denmark; 2 Rigshospitalet, Department of Pathology, Copenhagen, Denmark; 3 University of Copenhagen, Section of Biostatistics, Copenhagen, Denmark; 4 Statens Serum Institut, Department of Epidemiology Research, Copenhagen, Denmark; 5 Karolinska University Hospital, Department of Neurosurgery, Stockholm, Sweden; 6 Odense University Hospital, Department of Neurosurgery, Odense, Denmark; 7 University of Southern Denmark and BRIDGE, Clinical Institute, Odense, Denmark; 8 University of Copenhagen, Institute of Clinical Medicine, Copenhagen, Denmark

## Abstract

**Background:**

Malignant meningiomas comprise 2–5% of all meningiomas. The process of malignant transformation when benign meningiomas (WHO grade I-II) become malignant (WHO grade III) has not previously been investigated in sequential tumour surgeries. Upregulation of FOXM1 expression and DREAM-complex repression have shown phenotypical subgroups correlating with WHO grade and aggressiveness. We investigated the RNA expression of 30 genes central to meningioma biology and 770 genes involved in neuroinflammatory pathways in primary and secondary malignant meningioma patients who underwent one to several operations.

**Methods:**

We identified a cohort of consecutive malignant meningioma patients treated at Rigshospitalet, Copenhagen from 2000–2020 (n=51) and gathered their malignant tumours and previous WHO grade I/II tumours. The malignant cohort (MC) was counter matched with a benign cohort (BC) where patients had no recurrences during follow-up. RNA expression signatures from 140 samples from the MC and 51 samples from the BC were analysed with the Nanostring Neuroinflammation panel customized with 30 genes known to be relevant in meningioma phenotypes.

**Results:**

49% of MC patients had a previous grade I/II meningioma making them secondary malignant meningioma patients. Progression-free survival calculated from first malignant surgery to first recurrence or death showed no significant difference in the primary vs. secondary patients. Preliminary results of single-gene analysis of MC tumours showed FOXM1, MYBL2, TOP2A, BIRC5 expression was higher in WHO grade III samples. Gene-expression signatures in the individual patients and gene ontology enrichment analyses are in process.

**Conclusions:**

FOXM1, MYBL2, TOP2A, BIRC5 RNA expression levels seem to rise during malignant progression across patients. Gene-expression analysis using the Nanostring technology is feasible and a potentially powerful tool to distinguish meningiomas prone to malignant transformation from truly benign meningiomas.

